# Phase-sensitive optical time domain reflectometry based on geometric phase measurement

**DOI:** 10.1038/s41598-023-29972-4

**Published:** 2023-02-17

**Authors:** Sabahat Shaheen, Konstantin Hicke, Katerina Krebber

**Affiliations:** Bundesanstalt für Materialforschung und -prüfung (BAM), Unter den Eichen 87, 12205 Berlin, Germany

**Keywords:** Optics and photonics, Imaging and sensing

## Abstract

A phase-sensitive optical time domain reflectometer based on coherent heterodyne detection of geometric phase in the beat signal of light, is reported for the first time to our knowledge. The use of the geometric phase to extract strain makes it immune to polarisation diversity fading. This is because a polarisation mismatch between the interfering beams is not a hindrance to its measurement. The geometric phase is calculated using the amplitude of the beat signal and individual beam intensities without any need for phase unwrapping. It is measured per beat period and can be equated with the traditionally measured dynamic phase with appropriate scaling. The results show that the system based on the geometric phase successfully measures strain, free from polarisation mismatch fading and phase unwrapping errors, providing a completely novel solution to these problems.

## Introduction

Distributed fiber optic sensing based on Rayleigh scattering is used to detect dynamic vibrations along the length of an optical fiber with high sensitivity and long range, using the principle of phase-sensitive Optical Time Domain Reflectometry ($\phi$-OTDR)^1–4^. Thanks to the linear phase shifts experienced by light at the location of the vibration, a $\phi$-OTDR is able to reconstruct the vibration signal in amplitude, frequency and phase by phase demodulation of Rayleigh Backscatter (RBS) received from the optical fiber^1,5–7^. Thus, it finds useful applications in various fields such as seismology^8^, reservoir exploration^9^ and structural health monitoring^10^, to name a few. Phase demodulation refers to the extraction of spatio-temporal phase shifts in an optical fiber, typically performed using interferometric techniques^1^. A common technique is to use an imbalanced Mach-Zehnder interferometer to combine the backscatter signal with its delayed version to find the phase shifts^6^. Another important technique is to coherently hetero-^11,12^ or homodyne^13,14^ the RBS with a local oscillator (LO) to obtain a beat signal from which the phase information can be extracted^1^.

Coherent heterodyne detection for phase demodulation in a $\phi$-OTDR^11,12^ is an established technology that is commercially utilised for various ends because of its simple configuration and high signal-to-noise ratio^1^. Nonetheless, a major challenge to its performance is the phenomenon of signal fading^15^, further subdivided into two kinds. The first kind generally known as interference fading is a phenomenon of Rayleigh scattering itself. It is caused by non-uniform positions of scattering centers within the optical fiber and is a topic of active research^16,17^. The second kind, known as polarization mismatch fading or simply polarization fading, depends on interferometric conditions for coherent heterodyne detection^11^. That is, a mismatch between the State-of-Polarisation (SOP) of the two interfering beams leads to signal fades of arbitrary magnitudes, which is a hindrance in vibration detection for the duration of the fade. Such arbitrary and often large magnitudes may be raise false alarms in applications like Earthquake monitoring^18^. Several solutions have been proposed for polarisation fading, most of which are based on polarization diversity technique^19–24^. It is based on the probability that both the vertical and horizontal polarisations of the RBS may not be out of phase with the LO at the same time. Theoretically one can have a fading free signal if three SOPs separated by 90 degrees are used such that one of them will always be in phase with the LO^24^. Thus, a polarisation beam splitter is used to split the vertical and horizontal polarisations such that each is detected with a separate balanced photodetector, leading to hardware complexity and a larger data throughput^19^. Furthermore, phase unwrapping is an inherent feature of phase extraction from the complex amplitude of the beat signal, for which various noise sources may lead to unwrapping errors^25–27^.

Geometric Phase is generally studied in the space domain where the SOP of light can be rotated topologically using wave plates or a twisted optical fiber in the context of opto-electronic systems^28–31^. In fact, the use of geometric phase in optical fiber sensors^32^ or vibration sensing^33^ is not new. However, a novel context for the existence of geometric phase is in the time domain^34–36^ which has recently been studied in the beating of lightwaves. When two frequency offset beams of light interfere coherently, such as in coherent heterodyne detection, such that their respective SOPs are not identical, the SOP of the resulting beat signal oscillates in every beat period, just like its amplitude, thereby giving rise to geometric phase^36^. The term dynamic phase is used to refer to the remaining components of phase change^35^. The geometric phase is coupled to the dynamic phase, such that their sum remains constant equal to $\omega T \pm \pi$ in every beat period, *T*, $\omega$ being the angular frequency of light. Furthermore, it is a function of the relative intensities and the SOPs of the interfering beams^36^.

The geometric phase in a $\phi$-OTDR was recently measured by the authors^37^. The phase change traditionally measured in a $\phi$-OTDR^11^ is dynamic being caused by a change in length, refractive index or wavelength^38^. The measurement of the dynamic phase in a $\phi$-OTDR using coherent heterodyne detection requires the relative SOPs of the interfering beams to be identical^11^, in contrast, for the measurement of the geometric phase they must not be so^36^. In reality, the SOP of light in an optical fiber does not remain identical to the local oscillator beyond a few meters, yet matches enough for the phase difference to be measured over tens of kilometers^4,39^. The conditions of non-matching SOPs for the geometric phase imply that a $\phi$-OTDR based on coherent detection may benefit from using the geometric phase instead of the dynamic phase. Firstly, the polarisation mismatch fading may disappear as a mismatch of the relative SOP of the interfering beams is a condition not a hindrance for the existence geometric phase. Secondly, the calculation of the geometric phase does not require phase unwrapping and as a result does not suffer from unwrapping errors. In this work we replace the dynamic phase measured in a $\phi$-OTDR with the geometric phase. The performance of the new system is analysed and compared with that of a standard $\phi$-OTDR^12^.

## Theoretical background

The geometric phase, $\phi _g$, in the beat signal, $S_0$ of two interfering light beams, $S_0^{'}$ and $S_0^{''}$ is calculated as:^36^

1 $$\begin{aligned} \phi _g=\pm \pi -\sum \limits _{n=1}^{N}arg\left[S_0^{'} exp\left(\frac{-i\pi }{N}\right)+S_0^{''}exp\left(\frac{i\pi }{N}\right) +2\sqrt{S_0^{'}S_0^{''}}|\gamma _0|cos\left(\frac{n2\pi }{N}\right)\right] \end{aligned}$$

*N* is an integer number that represents the segments into which the period, *T* of the beat signal, $S_0$, has been divided. $\phi _g$ is calculated over each *T*. $\gamma _0$ in Eq. 1 is the normalised amplitude of $S_0$, calculated as follows^36^:

2 $$\begin{aligned} \gamma _0 = \frac{S_0}{\sqrt{S_0^{'}S_0^{''}}} \end{aligned}$$

Thus the calculation of $\phi _g$ involves the individual intensities of the interfering beams, $S_0^{'}$ and $S_0^{''}$, and the amplitude of the beat signal, $S_0$. With some modifications to a standard $\phi$-OTDR based on coherent heterodyne detection^12^, the geometric phase $\phi _g$ can be also be measured with it^37^. In the context of distributed fiber optic sensing $\phi _g$, being a function of relative intensities of the interfering beams as well as their relative polarisation states, may be used to devise new sensing mechanisms^37^. In this work, we use $\phi _g$ instead of the traditionally measured dynamic phase in a $\phi$-OTDR based on coherent heterodyne detection, to detect strain in an optical fiber in a distributed way. We accordingly refer to the new setup as $\phi _g$-OTDR.

As per Eq. 1, $\phi _g$ has a single value over one *T*, obtained after summing over *N* points . This is different for $\phi$ which is defined for every sample of the RBS. Thus, the spatial resolution of the $\phi _g$-OTDR is reduced by *N*. Thus, in the $\phi _g$-OTDR, the number of samples included in one gauge length, *G* are also reduced by *N*. $\phi$ is differentiated over *g*, i.e., the number of samples in one gauge length, *G*. Equivalently, $\phi _g$ is differentiated over $g_g$ given by:

3 $$\begin{aligned} g_g = g/N \end{aligned}$$

This limitation to spatial resolution can be overcome by taking a moving summation in Eq. 1. Moreover, *N* depends on the frequency offset between the interfering beams, $\Delta f$ and the sampling rate, $f_s$ at which $S_0^{'}$, $S_0^{''}$ and $S_0$ are sampled after conversion to respective electrical signals via photodetectors.

4 $$\begin{aligned} N = f_s/\Delta f \end{aligned}$$

The relationship between the two phases is given as:

5 $$\begin{aligned} \phi = -g\cdot {\phi _g} \end{aligned}$$

The minus sign in Eq. 5 indicates that $\phi _g$ is out of phase with $\phi$ due to their coupling in each beat period, i.e., when one increases, the other decreases^36^. The conversion from phase to strain, $\epsilon$ is achieved as follows^7^:

6 $$\begin{aligned} \epsilon = \frac{\lambda \Delta \phi }{4n\pi \xi G} \end{aligned}$$

where, $\Delta \phi$ is the differential phase derived from the standard setup or $\phi _g$-OTDR as per Eq. 5,$\xi$ and *n* are material dependent constants equal to 0.78 and 1.46, respectively for Silica glass while $\lambda$ is the wavelength of light. The gauge length, *G* is calculated as^38^:

7 $$\begin{aligned} G = v\tau /2 \end{aligned}$$

where *v* is the speed of light in the fiber and $\tau$ is the pulse width of the light signal used to interrogate the optical fiber. The spatial resolution of the system is calculated by dividing the samples per interrogation cycle over the fiber length in meters. *g* is calculated by multiplying the spatial resolution by *G*^38^.

## Experimental setup

**Figure 1:**
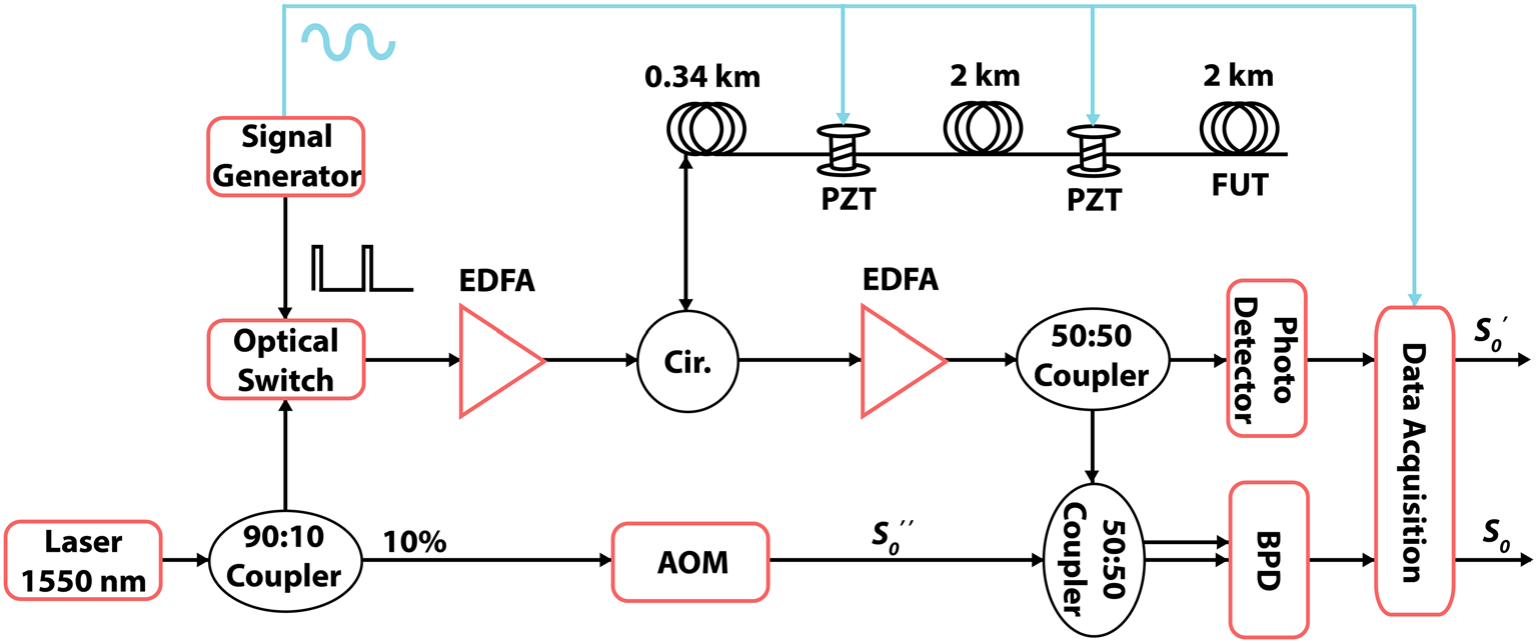
Block diagram of the $\phi _g$-OTDR setup. EDFA: erbium doped fiber amplifier, PZT: piezoelectric transducer, FUT: fiber-under-test, BPD: balanced photodetector, AOM: acousto-optic modulator, Cir.: 3-port optical circulator. Note that the cyan colour is associated with the sinusoidal test signal.

Figure 1 shows the hardware setup used to measure $\phi _g$^37^. The measurement of $\phi _g$ as per Eq. 1 requires the individual intensities of the interfering beams, $S_0^{'}$ and $S_0^{''}$, in addition to the beat signal^36^, later also required in the standard setup^12^. The individual beams in our case are the LO, $S_0^{''}$ and the RBS, $S_0^{'}$. Moreover, the branch carrying the RBS signal from the FUT is also called the probe branch. The LO branch serves as a reference and has a constant amplitude that is easily measured once using a power meter. To measure the intensity of the RBS, we split it into two parts using a 50:50 coupler such that half of the light is sent for direct detection using a single photodetector, thus giving us $S_0^{'}$, while the other half is sent for coherent heterodyning as in the standard setup.

Light from a narrow linewidth laser operating at 1550 nm wavelength is split in the ratio of 90:10 to be sent into the probe and LO branch respectively. In the probe branch, a high-speed polarization-dependent optical switch with a switching speed of 1 ns and an on/off ratio of 70 dB is employed to get an optical pulse train with a repetition frequency of 20 k. A pulsed Erbium-doped fiber amplifier (EDFA) then amplifies the pulses while suppressing any signal in the off state and sends them into the FUT via the transmission port of a 3-port optical circulator. RBS received via the reflection port of the circulator is amplified again using another EDFA and split equally using a 1×2 50:50 coupler. Half of the light is sent to a single photodetector, thus giving us $S_0^{'}$ to use in Eq. 1. The other half is sent to one of the input branches of a 2×2 50:50 coupler. The LO is given a frequency offset using an Acousto-optic Modulator (AOM) and then sent to the second branch of the 2×2 50:50 coupler. The latter interferes the frequency-shifted LO and pulsed RBS signal together and then splits the output equally between its two output ports. These two outputs are connected to a balanced photo-detector (BPD), which in turn downshifts the signal frequency giving us the beat signal, $S_0$, at the offset frequency of the AOM. The outputs of both photo-detectors are sampled at 500 M Samples/second using a high speed analog-to-digital converter (ADC). The signal generator provides the modulating signal at 20 kHz to the optical switch for pulse carving. It also provides the acoustic test signal provided to the PZTs. We tap this second input and connect it to the third port of the ADC whose digital output in Volts can be converted to the corresponding strain value experienced by the fiber on the PZT by its multiplication with an appropriate conversion factor. This provides us with a handy input signal that is used as a reference in some of the results shown below. The above setup can be used to extract the traditionally measured phase by taking the Hilbert transform of the beat signal $S_0$ and extracting the phase from its complex amplitude^12^. The same setup is also used to calculate $\phi _g$ by first normalising $S_0$ as per Eq. 2 and then finding its envelope, $|\gamma _0|$ also using Hilbert transform. $S_0^{'}$, the output of the single photo-detector and the constant $S_0^{''}$, measured using power meter are plugged into Eq. 1 along with $|\gamma _0|$ to calculate $\phi _g$ per beat period.

The samples per beat period, *N*, are calculated by dividing the sampling rate over the beat signal frequency, $\Delta f$, as per Eq. 4. Two different AOMs with $\Delta f$ equal to 40 MHz and 110 MHz were used, giving values of *N* equal to 12.5 and 4.5, respectively. As *N* is an integer as per Eq. 1, $\phi _g$ is calculated in both cases by summing over two beat periods to avoid rounding errors, giving $N=25$ and $N=9$, respectively for the two AOMs. $\phi _g$ is cumulative over the beat periods considered, therefore, if considering a single beat period, the value of $\phi _g$ is halved. The other way to change *N*, as per Eq. 4, is by changing the sampling rate of the ADC, $f_s$. This usually requires advanced handling of the ADC and may involve real-time system errors. It is noted that we use the AOMs only for frequency shifting. It is done to have the same rise-and-fall times for both AOMs because typically the rise-and-fall times of an AOM changes with its frequency offset. The hardware setup shown in Fig. 1 is used to interrogate a 4.5 km long fiber-under-test (FUT). Two piezoelectric transducer (PZT) cylinders are inserted in the FUT at approximately 0.345 and 2.35 km. The PZTs expand and contract in response to an electrical signal, known as the stimulus, which is applied by one of the outputs of the signal generator, thus incurring strain to the 15-meter-long fiber segments that are wound around them.

## Experimental results

**Figure 2:**
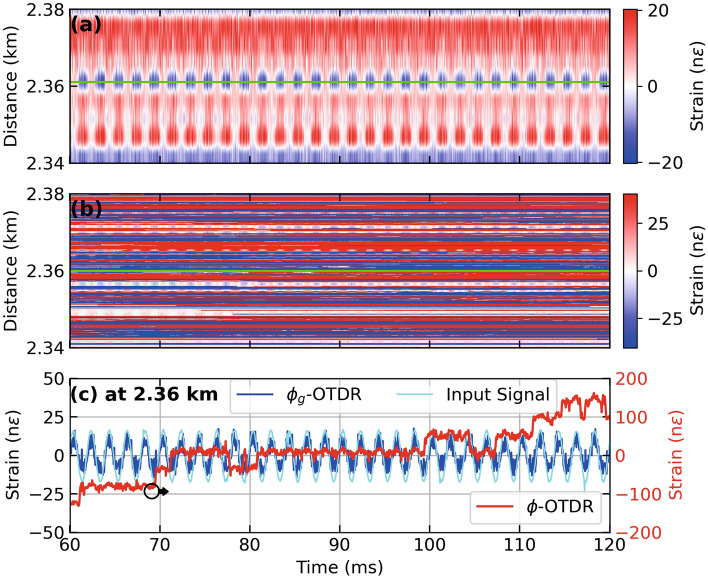
Strain calculated from dynamic and geometric phase, respectively, for a PZT centered at a fiber distance of 2.355 km driven by a sinusoidal test stimulus at 500 Hz giving (**a**) Distance-time contour plot of strain extracted from $\phi _g$-OTDR, (**b**) Distance-time contour plot of strain extracted from $\phi$-OTDR, (**c**) Time series of strain at 2.36 km (marked by green lines in (a) and (b)) extracted from dynamic phase (red lines, right axis also marked by an arrow), geometric phase (blue lines, left axis) in response to test stimulus (cyan lines, left axis), employing an AOM with a frequency offset of 40 MHz.

An AOM with a frequency offset of 40 MHz is used in the hardware setup shown in Fig. 1 to interrogate the FUT, a 15 meter section of which is wrapped around a PZT stimulated by a 500 Hz sinusoidal signal with peak strain of 20 n$\epsilon$ at a fiber distance of 2.355 km, with a 150 ns pulse. A pulse width of 150 ns gives $G = 15$ as per Eq. 7, $g = 75$ and $g_g=3$ as per Eq. 3 for this AOM. Figure 2 (a,b) show distance-time contour plots of strain calculated using $\phi _g$ and $\phi$, respectively. The strain stimulus is recovered by both systems in terms of frequency and amplitude. However, several arbitrary signal amplitudes attributable to polarisation mismatch fading and/or unwrapping errors can be marked in Fig. 2 (b) which are absent in Fig. 2 (a). Figure 2 (c) shows the strain extracted from the two systems as a time series at a fiber distance of 2.36 km where the the above mentioned effects can be appreciated in a time series of strain.

**Figure 3:**
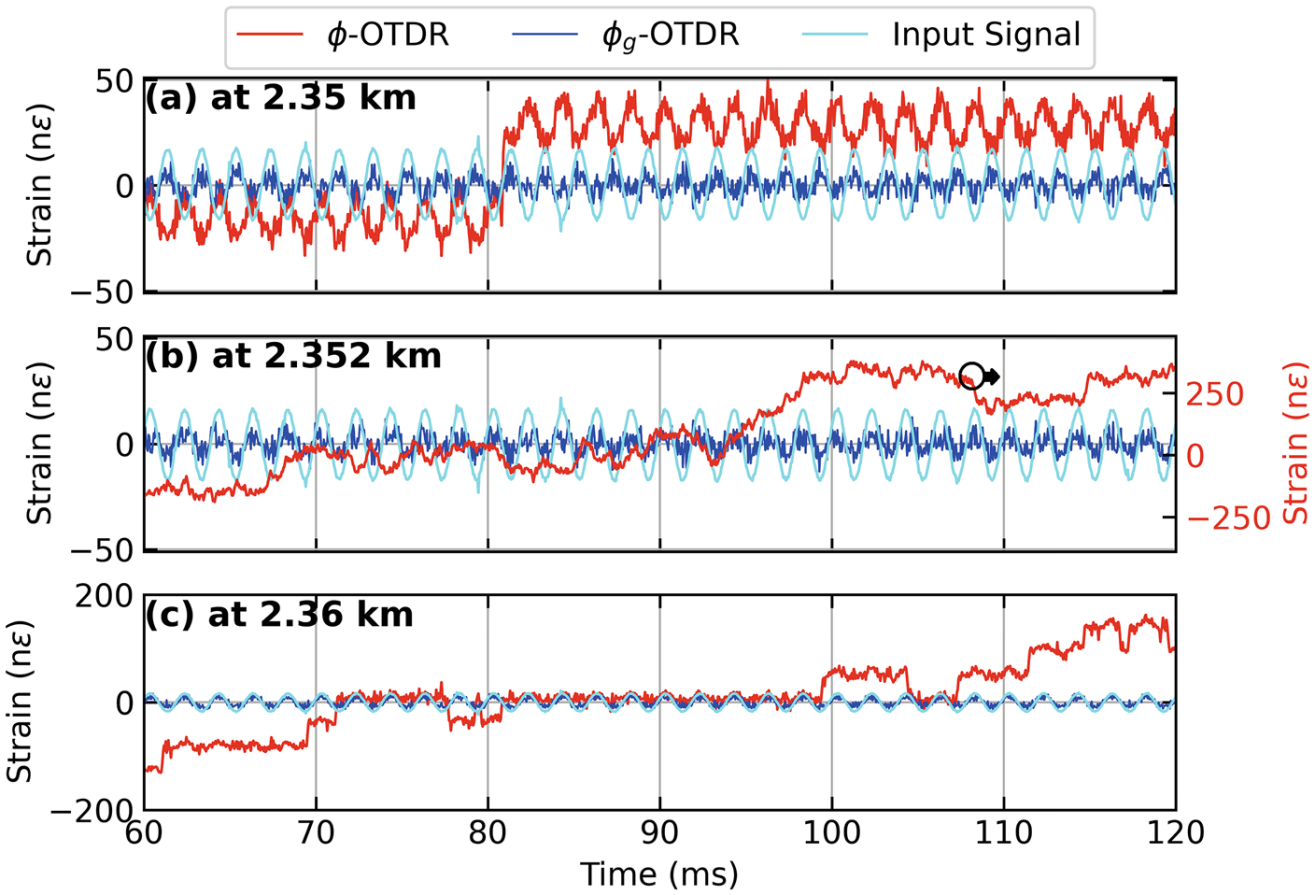
Strain calculated from dynamic phase (red lines) and geometric phase (blue lines), respectively, using an AOM with a frequency offset of 40 MHz for a sinusoidal test stimulus (cyan lines) at 500 Hz applied to a PZT inline the FUT giving (**a**) Time series of strain at 2.35 km, (**b**) Time series of strain at 2.352 km. Note the red lines in (**a**) correspond to the right axis as marked by an arrow. (**c**) Time series of strain at 2.36 km.

Figure 3 show the results from three different fiber distances from Fig. 2 (a,b) as time series. At a fiber distance of 2.35 km, as shown in Fig. 3 (a), the strain extracted from the dynamic phase (red lines) shows an unwrapping error, while the strain extracted from geometric phase (blue lines) appears immune from it . Figure 2 (b) shows a time series close to the center of the PZT at 2.352 km where $\phi _g$-OTDR reconstructs the input strain stimulus (cyan lines) with much more accuracy than the strain extracted from $\phi$-OTDR which suffers a polarization-mismatch fade. Figure 2 (c), the strains from both systems are compared at a fiber distance of 2.36 km. Here, the accuracy of the $\phi _g$-OTDR is highest as compared with Fig. 2 (a) and (b), while the strain extracted from $\phi$ is marred by several unwrapping errors.

**Figure 4:**
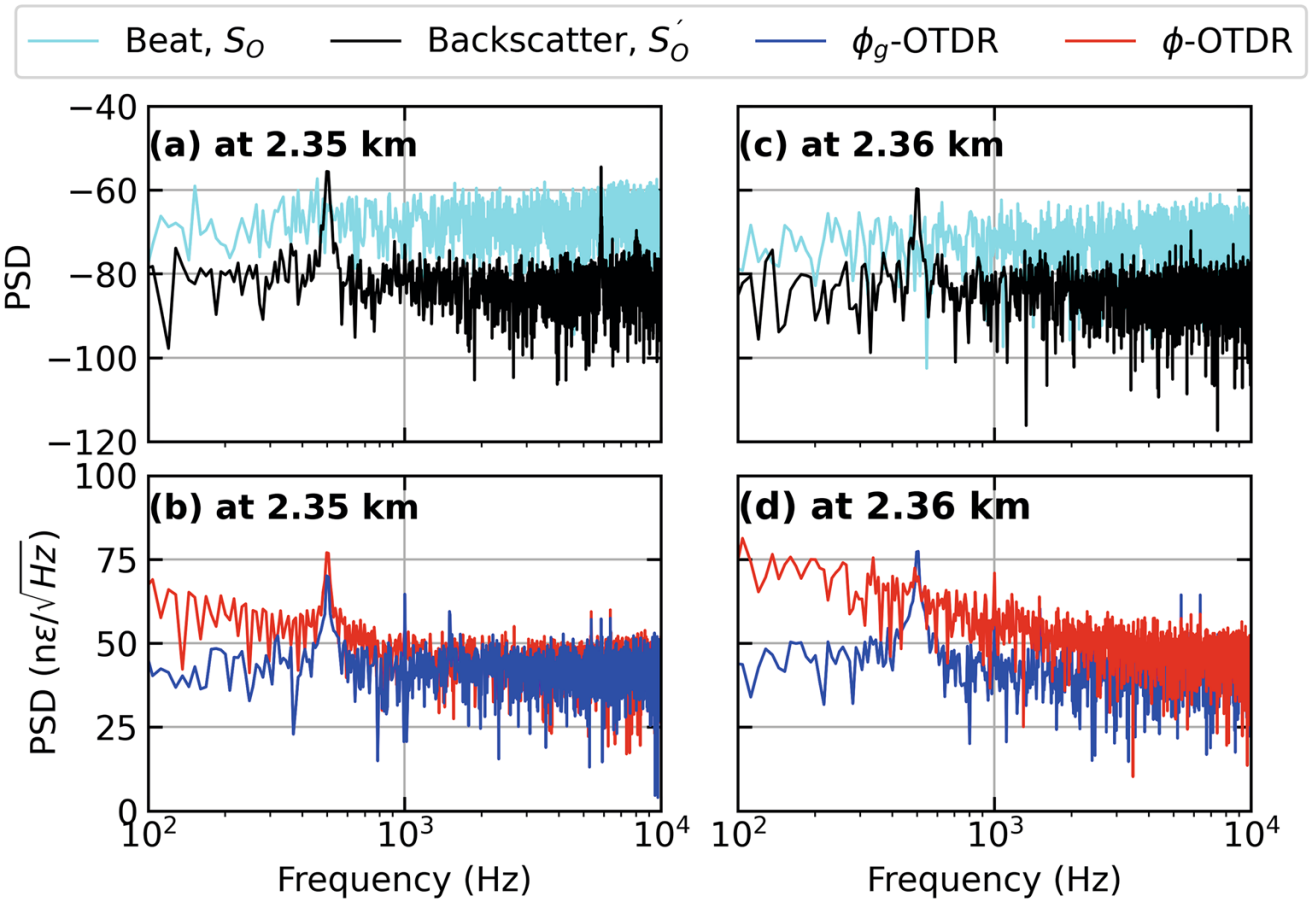
Power spectral density (PSD) of (**a**) beat signal, $S_0$ and Backscatter Intensity, $S_0^{'}$ at 2.35 km, (**b**) Strain calculated from dynamic and geometric phase, respectively at 2.35 km (**c**) Beat Signal, $S_0$ and Backscatter Intensity, $S_0^{'}$ at 2.36 km, (**d**) Strain calculated from dynamic and geometric phase, respectively at 2.36 km, for a PZT centered at a fiber distance of 2.355 km driven by a sinusoidal test stimulus at 500 Hz, employing an AOM with a frequency offset of 40 MHz.

In Fig. 4 (a,c), we look at the spectra of the beat signal, $S_0$ and backscatter signal, $S_0^{'}$ at a fiber distance of 2.35 km and 2.36 km respectively, using the same data as Figs. 2 and 3 . The corresponding strain values calculated from these signals using $\phi$ and $\phi _g$ are plotted in Fig. 4 (b,d), whose time series are already given in Fig. 3 (a,c). As shown in Fig. 4 (a), $S_0^{'}$ has an SNR of approximately 20 dB with higher order harmonics at 1.5 kHz, while $S_0$ has a signal strength of around –50 dB. Figure 4 (b) shows that the SNR of strain calculated from both systems is almost the same at 20 dB. However, the one from $\phi$ a higher noise floor, especially at lower frequencies upto 1 kHz. It is also noted that strain calculated using $\phi _g$ has more higher order harmonics. Figure 4 (c) shows that at a fiber distance of 2.36 km, the SNR of $S_0^{'}$ is slightly lower but the higher order harmonic has disappeared. Figure 4 (d) shows that the strain calculated from $\phi$ is completely distorted due to the signal fade (shown in Fig. 3 (c) as a time series). Whereas, the strain calculated from $\phi _g$ has an SNR of around 25 dB but also contains higher-order harmonics. Overall, the strain calculated by $\phi$ is more accurate at 2.35 km while that calculated from $\phi _g$ performs equally well at 2.36 km. This can be attributable to polarisation states of the interfering beams at the respective fiber distances. If we compare these best-case performances of $\phi$- and $\phi _g$-OTDR at 2.35 and 2.36 km, respectively, they perform equally well in terms of SNR. In comparison, the former has a higher noise floor at lower frequences upto 1 kHz while the later suffers from more higher order harmonics compared with the former.

**Figure 5:**
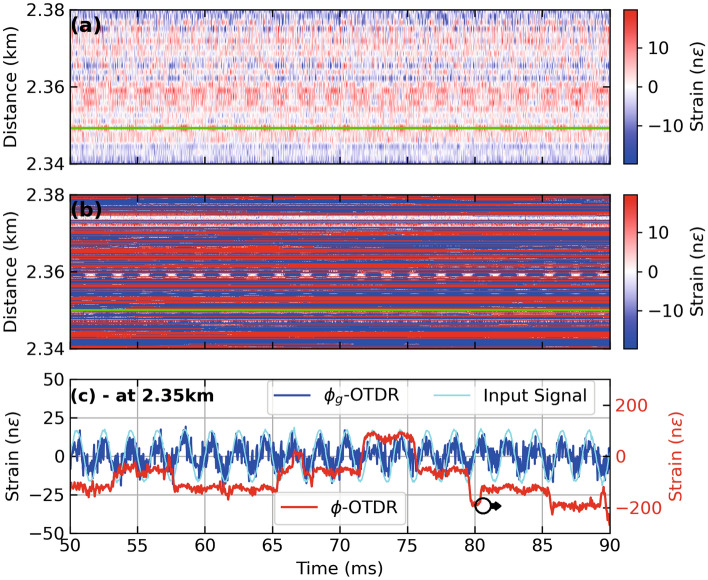
Strain calculated from dynamic and geometric phase, respectively, for a PZT centered at a fiber distance of 2.355 km driven by a sinusoidal test stimulus at 500 Hz giving (**a**) Distance-time contour plot of strain extracted from $\phi _g$-OTDR, (**b**) Distance-time contour plot of strain extracted from $\phi$-OTDR and (**c**) Time series of strain at 2.35 km [marked by green lines in (a) and (b)] extracted from dynamic phase (red lines, right axis also marked by an arrow), geometric phase (blue lines, left axis) in response to test stimulus (cyan lines, left axis), employing an AOM with a frequency offset of 110 MHz.

The results shown in Fig. 5 are obtained by repeating the test scenario of Fig. 2 with the second AOM with $\Delta f = 110$ MHz utilising a 100 ns pulse. A pulse width of 100 ns gives $G = 10$ as per Eq. 7, $g = 50$ and $g_g=1$ as per Eq. 3, for this AOM. In this case, the same strain stimulus at 500 Hz with a peak value of 20 n$\epsilon$ is applied to a 15 meter section of FUT wrapped around a PZT centered at 2.36 km. In Fig. 5 (a), the strain signal is extracted using $\phi _g$ while in Fig. 5 (b) it is extracted from $\phi$. Their comparison shows that the later reconstructs the signal with several high-amplitude fades attributable to polarization mismatches. Horizontal green lines in Fig. 2 (a) and (b) mark the fiber distance at which the time series in Fig. 5 (c) is shown. In Fig. 2 (c), data is sliced at a fiber distance of 2.35 km and shown as a time series comparing the input stimulus (cyan lines), strain extracted from $\phi$ (red lines, right axis) and that from $\phi$ (blue lines, left axis). Signal extracted from $\phi _g$ shows immunity to a polarization mismatch and unwrapping errors which the signal extracted from $\phi$ is suffering from. Strain extracted from $\phi _g$ in Fig. 2 (c) has a lower spatial resolution as compared with the one shown here in Fig. 5 (c). This is due to lower value of *N* for teh AOM with an offset $\Delta f = 40$ MHz. On the other hand, a higher *N* in Fig. 2 (c) gives better results in terms of amplitude reconstruction of the strain stimulus, notwithstanding the slightly larger pulse width used there.

**Figure 6:**
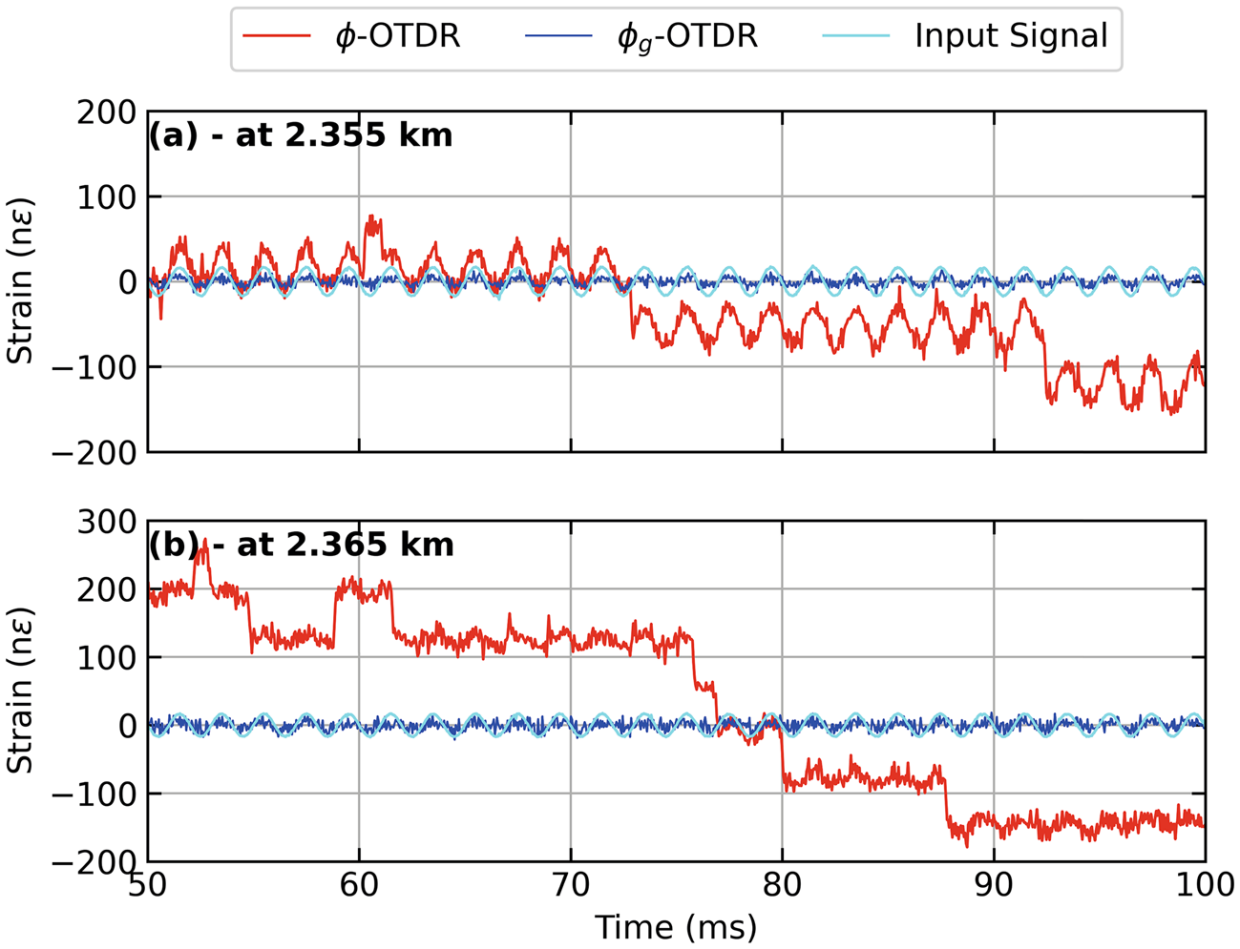
Strain calculated from dynamic phase (red lines) and geometric phase (blue lines), respectively, using an AOM with a frequency offset of 110 MHz for a sinusoidal test stimulus (cyan lines) at 500 Hz applied to a PZT centered at 2.36 km inline the FUT, giving (**a**) Time series of strain at 2.355 km, (**b**) Time series of strain at 2.365 km.

Unwrapping errors may occur in in coherent heterodyne detection $\phi$-OTDR due to noise and/or a polarisation mismatch^25^. In this context, the advantage of using $\phi _g$ is demonstrated in Fig. 6 (a) and (b) where strain extracted from $\phi _g$ and $\phi$ is compared. A PZT at a distance of 2.35 km is stimulated with a 500 Hz sinusoid stimulus; interrogating with a pulse width of 100 ns giving a peak value of strain equal to approximately 20 n$\epsilon$. In Fig. 6 (a), at a fiber distance of 2.355 km, the effect of both phase unwrapping errors and polarisation mismatch is seen on the strain extracted from the dynamic phase (red lines) while the strain extracted from $\phi _g$ shows robustness towards both of these problems. Similarly, in Fig. 6 (b), at a fiber distance of 2.365 km, the condition of strain extracted from $\phi$ (red lines) has become even worse while the one extracted from $\phi _g$ remains the same.

**Figure 7:**
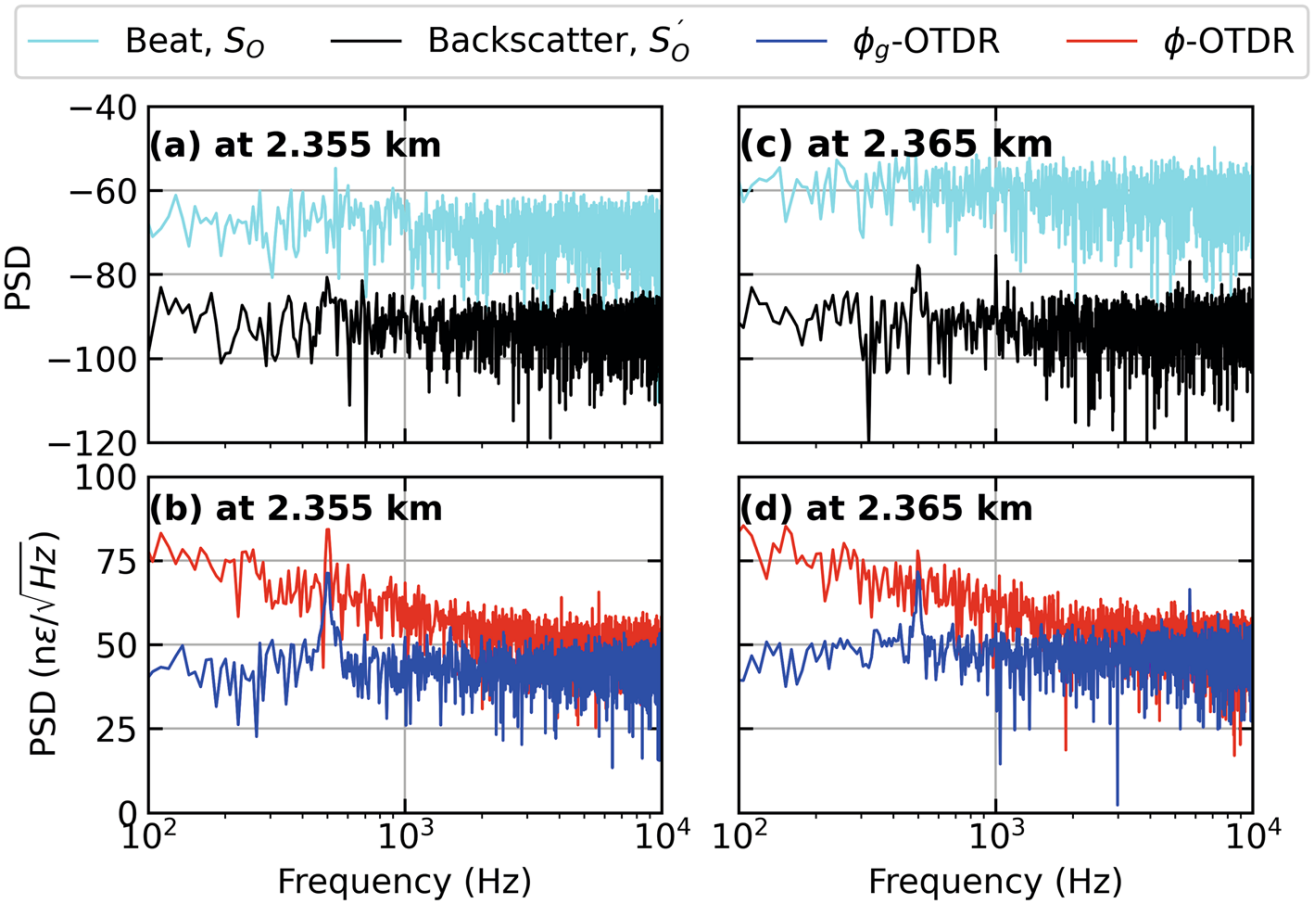
Power spectral density (PSD) of (**a**) beat signal, $S_0$ and Backscatter Intensity, $S_0^{'}$ at 2.355 km, (**b**) Strain calculated from dynamic and geometric phase, respectively at 2.35 km (**c**) Beat Signal, $S_0$ and Backscatter Intensity, $S_0^{'}$ at 2.365 km, (**d**) Strain calculated from dynamic and geometric phase, respectively at 2.365 km, for a PZT centered at a fiber distance of 2.36 km driven by a sinusoidal test stimulus at 500 Hz, employing an AOM with a frequency offset of 40 MHz.

In Fig. 7 (a) and (c), we look at the spectra of the beat signal, $S_0$ and backscatter signal, $S_0^{'}$ at a fiber distance of 2.355 km and 2.365 km respectively. The corresponding strain values calculated from these signals using $\phi$ and $\phi _g$ are plotted in Fig. 7 (b,d), whose time series are given in Fig. 6 (a,b). As shown in Fig. 7 (a), $S_0^{'}$ has a very low SNR of a few dBs with a higher order harmonic at 1.5 kHz, while $S_0$ has a signal strength of around –50 dB. Figure 7 (b) shows that the strain calculated using $\phi _g$ has an SNR of 12 dB while the one from $\phi$ is slightly lower at round 10 dB. The later has a higher noise floor at all frequencies. Figure 7 (c) shows that at a fiber distance of 2.365 km, the SNR of $S_0^{'}$ is around 10 dBs and higher order harmonics at 1 and 1.5 kHz. Figure 7 (d) shows that the strain calculated from $\phi$ is distorted due to the signal fade (shown in Fig. 6 (b) as a time series). Whereas, the strain calculated from $\phi _g$ has an SNR of around 12 dB and contains a higher order harmonic at 1.5 kHz. Overall, the strain calculated by $\phi$ is quite similar at 2.355 and 2.365 km with only the addition of higher order harmonic at 2.365 km. In comparison, the strain from $\phi _g$ is performing well at 2.355 km with a signal power around 5 dB higher than the $\phi _g$-OTDR but at 2.365 km, when it encounters a fade, its performance is severely distorted.

**Figure 8:**
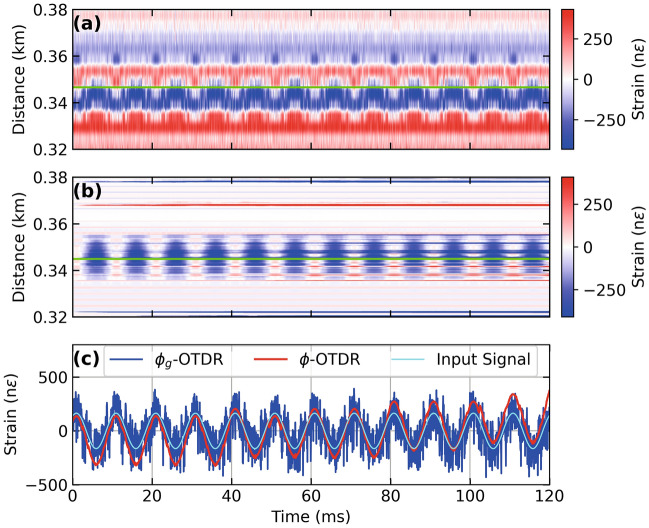
Strain calculated from dynamic and geometric phase, respectively, for a PZT at fiber distance of 0.34 km driven by a sinusoidal test stimulus of 200 n$\epsilon$ and 100 Hz giving, (**a**) Distance-time contour plot of strain extracted from $\phi _g$-OTDR, (**b**) Distance-time contour plot of strain extracted from $\phi$-OTDR and (**c**) Time series of strain at a fiber distance of 0.345 km [marked by green lines in (a) and (b)] extracted from dynamic phase (red lines), geometric phase (blue lines, left axis) in response to test stimulus (cyan lines, left axis), employing an AOM with a frequency offset of 40 MHz.

**Figure 9:**
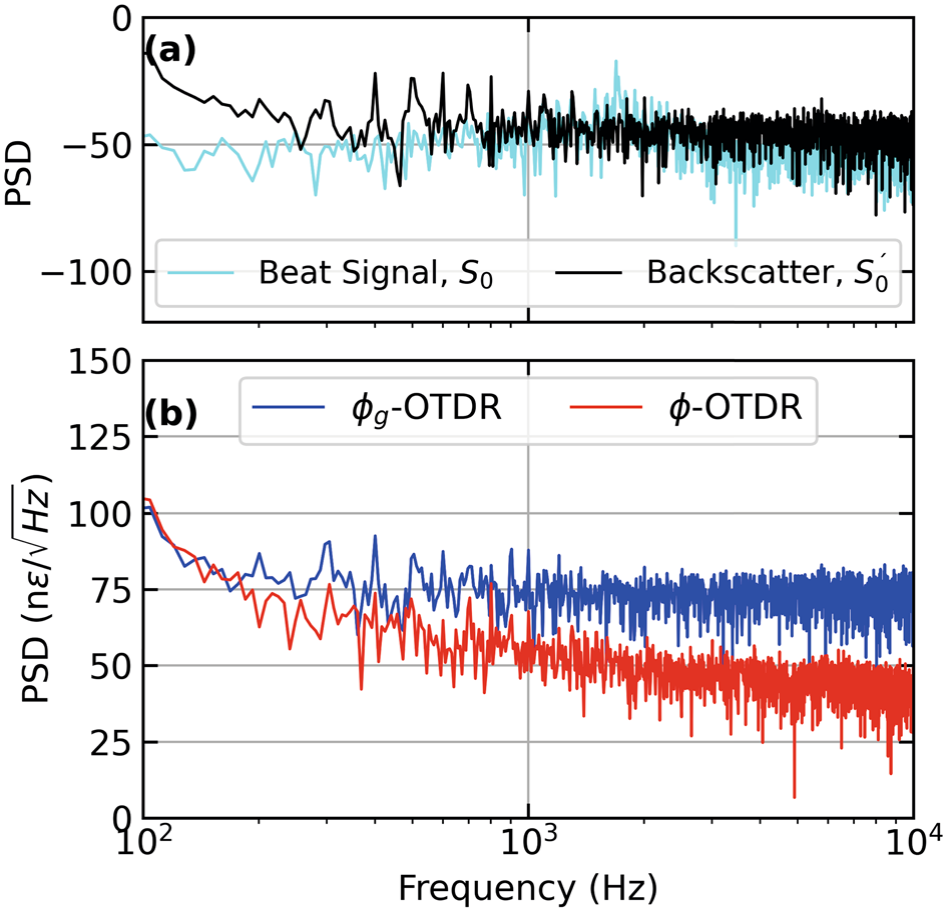
Power Spectral Density (PSD) of (**a**) Beat Signal, $S_0$ and Backscatter Intensity, $S_0^{'}$ at 2.345 km, (**b**) Strain calculated from dynamic and geometric phase, respectively at 0.345 km, for a PZT centered at a fiber distance of 2.36 km driven by a sinusoidal test stimulus at 500 Hz, employing an AOM with a frequency offset of 40 MHz.

In Fig. 8, we look at the dynamic range of the $\phi _g$-OTDR as compared to $\phi$-OTDR based on coherernt heterodyne detection. Testing scenario for Fig. 2 and 3 utilising 40 MHz frequency offset AOM is replicated with the difference that the strain stimulus is a 100 Hz sinusoid with an amplitude of approximately 200 n$\epsilon$ and the PZT is centered at 0.345 km. A pulse width of 100 ns gives $G = 10$ as per Eq. 7, $g = 50$ and $g_g=2$ as per Eq. 3 for this AOM. Fig. 8 (a,b) compare the distance-time contour plots of strain obtained from $\phi _g$-OTDR and $\phi$-OTDR, respectively. Horizontal green lines in Fig. 8 (a) and (b) mark the fiber distance at which the time series in Fig. 8 (c) is shown. Fig. 8 (c) compares the time series of strain at a fiber distance of 0.345 km obtained from the two systems shown in Fig. 8 (a) and (b). The input strain stimulus (cyan lines) can no longer be faithfully reproduced by any of the two systems as the dynamic range has been crossed. We can see that the strain obtained from $\phi _g$-OTDR (blue lines) shows a similar trend to the one obtained from $\phi$-OTDR (red lines). This signal distortion due to exceeding the dynamic range can be better seen in Fig. 9.

In Fig. 9 (a), we look at the spectra of the beat signal, $S_0$ and backscatter signal, $S_0^{'}$ at a fiber distance of 2.345 km. The corresponding strain values calculated from these signals using $\phi$ and $\phi _g$ are plotted in Fig. 9 (b), whose time series are given in Fig. 8 (c). As shown in Fig. 9 (a), $S_0^{'}$ teh actual SNR is approximately 25 dB but it is buries in the equally high higher-order harmonics. The $S_0$ has a signal strength of around -50 dB and is itself modulated at a frequency of 1.1 kHz. Figure 9 (b) shows that the strain calculated using $\phi _g$ has an SNR of approximately 18 dB while the signal from $\phi$ has an SNR of 5 dB but it is also engulfed in noise and higher order harmonics. The later has a much lower noise floor at higher frequencies.

## Discussion

The presented results show that $\phi _g$ contains a higher number of high-frequency spikes as compared with $\phi$, when there are no fades or unwrapping errors in the later. The source of this noise may be the changing polarisation states of the interfering beams. Another reason may be the splitting of the backscatter signal, into two equal parts via a 50:50 coupler. Theoretically, the directly detected signal, $S_0^{'}$ and its counterpart sent for coherent heterodyning are the same signal. However, in reality they are being detected by two different photodetectors and this may become a source of noise given the varying photoelectric coefficients of the respective detectors. In future work, an effort to have this factor reduced by appropriate choice of photodetectors will be made. Nonetheless, due to its immunity from polarisation fading and unwrapping errors, the strain data acquired from $\phi _g$-OTDR requires much less post-processing for noise cancellation as compared with the $\phi$-OTDR.

Two methods for the measurement of the geometric phase were presented in^36^, such that, one relies on the relative intensities of the interfering beams and the amplitude of the beat signal while the other involves a Stokes receiver. We chose the first method because of its hardware simplicity in order to measure geometric phase in the beat signal of a $\phi$-OTDR based on coherent heterodyne detection^37^. However, this method relies on dividing the beat period into *N* samples and then numerically summing over them. In this regard, the seminal study that was carried out in free space only used two beams with constant amplitude and an $N = 200$ points^36^. Our earlier work^37^ reports an experiment that was carried out in an optical fiber medium, which is less noisy than free space, measuring the geometric phase even with $N=2$, while the current work shows that the differential geometric phase may be measured with a value of *N* as low as 9. Nonetheless, a higher value of $N=25$ gives better results.

Keeping the sampling rate fixed at 500 MSa/s, *N* can be changed by either changing the AOM offset frequency or by summing over a larger *N* in the digital domain. The first method is more robust as the average value of geometric phase in each beat period is closer to the actual value. We used two different AOMs with offsets of 110 and 40 MHz, giving us 4.5 and 12.5 samples per beat period, respectively. It might be ideal to have the sampling rate over the AOM offset frequency an integer number so $\phi _g$ can be calculated over a single beat period without having any numerical residues. Moreover, the $\Delta f$ of the AOM may be further reduced to increase *N*, giving us an $\phi _{g}$ closer to the actual strain value. On the other hand, having more samples per beat period leads to lower spatial resolution. In this regards, the method involving two stokes receiver given in^36^, even though very complex from the point of view of hardware, is worth exploring.

Considering the hardware complexity of the standard setup, our proposed measurement method has the downside that an additional single photodetector and ADC channel is utilised, doubling the data throughput. However, compared with the polarisation diversity technique for overcoming polarisation-mismatch fading, which utilises one or two extra balanced photodetectors as well as polarisation beam splitters and polarisers^19,21^ or the other phase demodulation technique involving three photodetectors^6^, our setup is relatively less complex. A final note about the measurement method is that it is imperative to use a narrow linewidth laser as the coherence of light plays a critical role in the detection of $\phi _g$^36^. It is also true for the traditional $\phi$-OTDR based on coherent detection as well^40^.

Our measurement setup enables us to compare the system performance for both OTDRs using the same hardware setup. On one hand this makes the comparison very fair being subject to the exact same conditions, on the other hand it presents an opportunity to use both systems in conjunction. This is doubly attractive because both systems have complimentary assumption of the relative SOPs of the interfering beams. Thus, when one system degrades, the other is bound to perform better. It is in fact possible to get a polarisation ’match’ fading, where the two beams have exactly the same SOP, however, the probability of it happening its very low. This is because the SOP of the interfering beams after a few meters ceases to be identical and its effect is carried forward till the end of the fiber^39^. It is further noted that even though the $\phi _g$-OTDR can measure the phase regardless of a polarisation mismatch, it can still suffer from unideal channel conditions. Thus, a system utilizing both phases in conjunction may benefit from the best of both.

## Conclusion

Newly reported geometric phase in the beat signal of two frequency offset beams is coupled to the dynamic phase in every beat period^36^. The measurement of geometric phase does not require the two interfering beams to have the same SOP as the traditional coherent detection does. Our results verify that a $\phi _g$-OTDR system based on geometric phase shows immunity to polarisation mismatch fading as well as unwrapping errors. Thus it provides a paradigm shift to the problem of polarisation fading, whose current solutions are mostly based on polarisation diversity detection^19–24^. The results are applicable to fiber interferometry based on heterodyne detection in general, thus, also applicable to coherent optical communication. Finally it is noted that the proposed solution is particularly suited to earthquake monitoring because the the phase unwrapping errors as well as polarisation mismatch can raise a false alarm. Moreover, the calculation of geometric phase per beat period instead of at every sample point results in fewer data points along the spatial axis, making it easier to process large volumes of monitoring data.

## Data Availability

Data underlying the results presented in this paper are available on request from S.S.
